# Efficient Construction of an Inverted Minimal H1 Promoter Driven siRNA Expression Cassette: Facilitation of Promoter and siRNA Sequence Exchange

**DOI:** 10.1371/journal.pone.0000767

**Published:** 2007-08-22

**Authors:** Hoorig Nassanian, Ana M. Sanchez, Alice Lo, Kenneth A. Bradley, Benhur Lee

**Affiliations:** 1 Department of Microbiology, Immunology and Molecular Genetics, David Geffen School of Medicine, University of California at Los Angeles, Los Angeles, California, United States of America; 2 Department of Pathology and Laboratory Medicine, David Geffen School of Medicine, University of California at Los Angeles, Los Angeles, California, United States of America; 3 University of California at Los Angeles AIDS Institute, David Geffen School of Medicine, University of California at Los Angeles, Los Angeles, California, United States of America; National Institute on Aging, United States of America

## Abstract

**Background:**

RNA interference (RNAi), mediated by small interfering RNA (siRNA), is an effective method used to silence gene expression at the post-transcriptional level. Upon introduction into target cells, siRNAs incorporate into the RNA-induced silencing complex (RISC). The antisense strand of the siRNA duplex then “guides” the RISC to the homologous mRNA, leading to target degradation and gene silencing. In recent years, various vector-based siRNA expression systems have been developed which utilize opposing polymerase III promoters to independently drive expression of the sense and antisense strands of the siRNA duplex from the same template.

**Principal Findings:**

We show here the use of a ligase chain reaction (LCR) to develop a new vector system called pInv-H1 in which a DNA sequence encoding a specific siRNA is placed between two inverted minimal human H1 promoters (∼100 bp each). Expression of functional siRNAs from this construct has led to efficient silencing of both reporter and endogenous genes. Furthermore, the inverted H1 promoter-siRNA expression cassette was used to generate a retrovirus vector capable of transducing and silencing expression of the targeted protein by>80% in target cells.

**Conclusions:**

The unique design of this construct allows for the efficient exchange of siRNA sequences by the directional cloning of short oligonucleotides via asymmetric restriction sites. This provides a convenient way to test the functionality of different siRNA sequences. Delivery of the siRNA cassette by retroviral transduction suggests that a single copy of the siRNA expression cassette efficiently knocks down gene expression at the protein level. We note that this vector system can potentially be used to generate a random siRNA library. The flexibility of the ligase chain reaction suggests that additional control elements can easily be introduced into this siRNA expression cassette.

## Introduction

The use of RNA interference as a method of gene silencing has been an effective tool in analyzing gene function. First discovered in *Caenorhabditis elegans* [1], the phenomenon of RNAi has also been observed in *Drosophila* [2], [3], *Trypanosoma* [4], and other organisms. RNAi also occurs in plants by a process called post-transcriptional gene silencing (PTGS) [5], [6] and in fungi by a process called quelling [7]. In mammalian cells, dsRNA longer than 30 nucleotides (nt) induces an interferon response [8] that triggers non-specific degradation of mRNA. As a result, the application of RNAi in mammalian cells has been limited. However, after Elbashir et al. showed that base-paired 21-22 nt siRNAs with 3′ overhanging ends were capable of mediating sequence-specific degradation of mRNA in *Drosophila* embryo lysates [9], they determined that siRNAs also mediated sequence-specific gene silencing in mammals [8] without induction of the interferon response. Thus with the identification of siRNAs as the mediators of RNAi, many siRNA expression systems have been developed for efficient gene silencing.

Plasmid-based expression systems that use type III RNA polymerase III (pol III) promoters such as the U6 promoter and the H1 promoter have been developed by several groups and used to express functional siRNAs [10]–[14]. In the type I and type II classes of pol III promoters, the promoter elements are located entirely within the transcribed region [15]. In the case of the type III class of promoters, transcription is driven by *cis*-acting elements that are found only in the 5′ region flanking the gene to be transcribed. It has been demonstrated that deletion of sequences that are downstream of the transcriptional start site in the mouse and human U6 promoters has no effect on transcription levels [15]. Being type III RNA pol III promoters, the U6 and H1 promoters share similar regulatory elements, although the H1 promoter is considered to be more compact [16].

The transcription of short hairpin RNAs (shRNAs) from plasmids using a U6 or H1 promoter gives rise to a 21-22 nt shRNA where the complementary strands are separated by a short loop sequence [10], [17], [18]. The loop sequence is cleaved by Dicer, an RNase III enzyme, producing the mature, 21-22 nt siRNA. Another approach in expressing siRNAs has been to use tandem pol III promoters to drive expression of the sense and antisense strands of the siRNA, which after being transcribed, will base pair and form a functional siRNA [11]. Inverted promoter systems that can drive transcription of the sense and antisense strands from the same piece of double stranded DNA encoding the cognate siRNA have also been developed. These systems include those using two U6 promoters [12], a mouse U6 promoter and human H1 promoter [13], and a human U6 promoter and human H1 promoter [14]. These systems have proven to be effective gene silencers when transfected into cells and there is some suggestion that siRNA expression from inverted promoters can lead to more efficient gene silencing than shRNA from single pol III promoters [14].

We report here the construction and function of pInv-H1, a plasmid-based siRNA expression system that uses two inverted minimal H1 promoters to express siRNAs. The H1 promoter used here consists only of the core 100 bp that has been previously shown to contain the full promoter activity [16]. Unique restriction sites flanking the central siRNA-encoding region were designed to facilitate downstream cloning of any siRNA sequence. Importantly, the entire expression cassette was generated through a ligase chain reaction (LCR) since the essential H1 promoter elements and the siRNA-encoding region were only about 257 bp in length. Furthermore, we show that the expression cassette can be moved into a retroviral expression vector, resulting in stable efficient expression of the siRNA in transduced cells and reducing expression of the target protein by greater than 80%. Such an inverted promoter system also opens the possibility of generating a random siRNA library. In addition, the flexibility of the construction of the LCR allows for future “promoter bashing” experiments to further improve upon the efficacy of pol III promoters.

## Results

### Construction of the inverted H1 promoter-siRNA expression construct, pInv-H1

**Figure 1:**
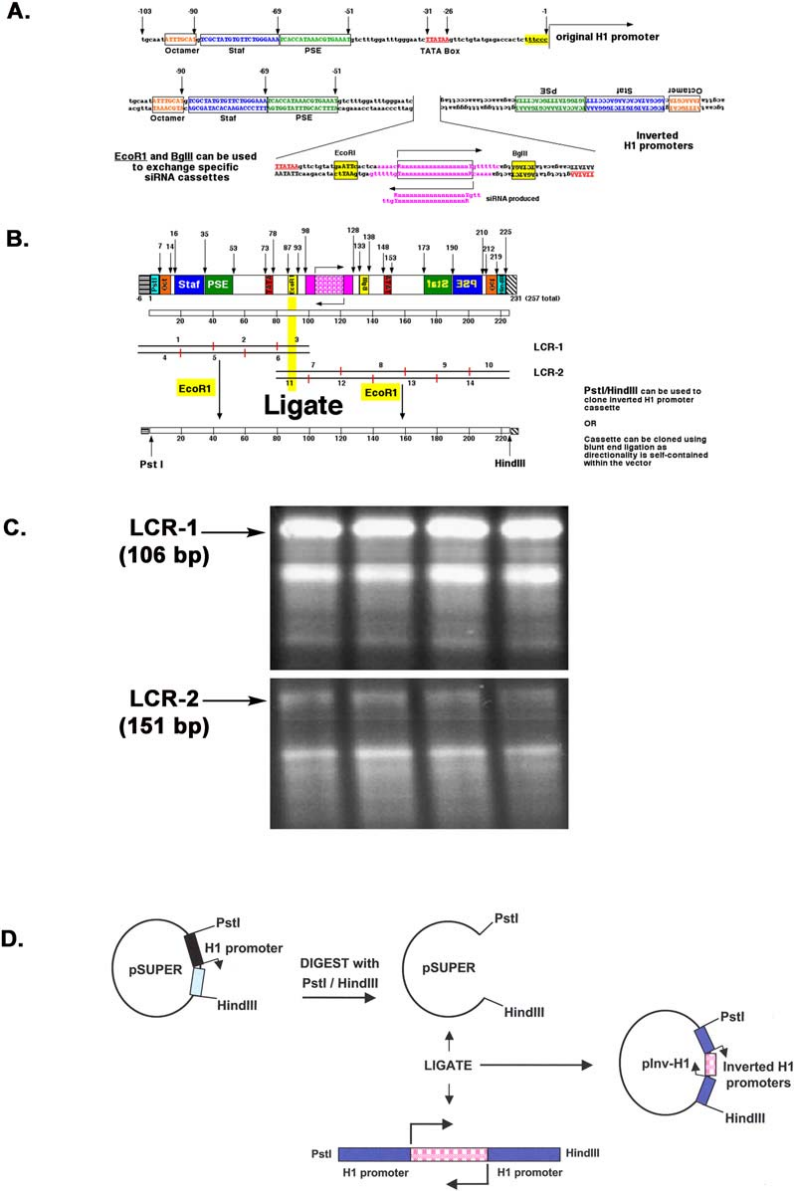
Construction of pInv-H1. A. Organization of the inverted H1 promoters and sequence encoding a specific siRNA. The expression cassette contains essential promoter elements. The *Eco*RI and *Bgl*II sites flank the siRNA sequence and can be used to exchange siRNA cassettes. B. Generation of the Inv-H1-siRNA cassette by a ligase chain reaction (LCR). The numbers associated with the LCR-1 and LCR-2 products correspond to individual oligonucleotides that are ligated together. Digesting LCR-1 and LCR-2 with *Eco*RI allows for ligation and formation of the full-length product that can be cut with *Pst*I and *Hind*III and cloned into pSUPER. C. Gel electrophoresis of LCR-1 and LCR-2 products. Ten microliters of the LCR reaction were run in each lane of a 3% Metaphor agarose gel. The fragments corresponding to 106 bp (LCR-1) and 151 bp (LCR-2) were gel purified. D. Cloning scheme used to generate pInv-H1. The pSUPER construct and the LCR-derived product were digested with *Pst*I and *Hind*III and ligated together. The original full-length H1 promoter driving expression of the shRNA product in pSUPER has now been replaced with the inverted H1 promoter siRNA cassette.

The pInv-H1 construct uses two inverted minimal human H1 promoters to independently transcribe the sense and antisense strands of the siRNA which will anneal to form a functional siRNA duplex. Figure 1 illustrates the general strategy used for assembling this inverted H1 promoter vector. Although the H1 promoter is ∼300 bp in length, only four promoter elements lying within 100 bp of 5′ flanking sequences have been shown to be essential for maximal *in vitro* and *in vivo* expression, making the H1 promoter a compact promoter [16]. These four elements include the octamer motif, the Staf-binding site, the proximal sequence element (PSE) and the TATA box. Seeing that these four elements are enough for maximal promoter activity, we used this minimal H1 promoter in our pInv-H1 construct (Fig. 1A).

The inverted H1 promoter-siRNA expression cassette was generated through a ligase chain reaction (LCR) in which several short, overlapping oligonucleotides were annealed and ligated at a high temperature to form a full-length gene [19]. The expression cassette was formed through two separate LCR reactions, generating the LCR-1 and LCR-2 products (Fig. 1B–C). The LCR-1 product (106 bp) was made by ligating six overlapping oligonucleotides and contains the sequence of the forward H1 promoter (Fig. 1B). The 5′ end of this sequence contains a *Pst*I site and the 3′ end contains an *Eco*RI site. The LCR-2 product (151 bp) was generated by ligating eight overlapping oligonucleotides and contains the 19 nt siRNA-encoding sequence and the inverted H1 promoter sequence (Fig. 1B). The 5′ end of this sequence contains an *Eco*RI site and the 3′ end contains a *Hind*III site. Since the LCR method relies on the efficient ligation of several overlapping oligonucleotides, incompletely synthesized products often appear as smaller fragments, but the full-length products, LCR-1 and LCR-2, can always be easily detected (Fig. 1C). After the LCR-1 and LCR-2 products were independently assembled, they were individually cut with *Eco*RI and then ligated to form the full-length Inv-H1-siRNA cassette (Fig. 1B). The full-length cassette (257 bp) was then cut with *Pst*I and *Hind*III, which flanks the entire inverted siRNA expression cassette, and cloned into the corresponding *Pst*I/*Hind*III sites of the pSUPER vector [10] (Fig. 1D). Although the original pInv-H1 construct was made with the siRNA sequence targeting the mRNA of DC-SIGN, a C-type lectin receptor highly expressed on dendritic cells and involved in binding to ICAM-2 [20], ICAM-3 [21], and HIV-1 gp120 [22], [23], the oligonucleotides were designed with *Eco*RI and *Bgl*II sites flanking the siRNA sequence, allowing for the convenient exchange of siRNA sequences. Thus, we also generated a pInv-H1 construct capable of giving rise to a siRNA targeting the p53 mRNA.

### pInv-H1 efficiently silences expression of DC-SIGN and p53

**Figure 2:**
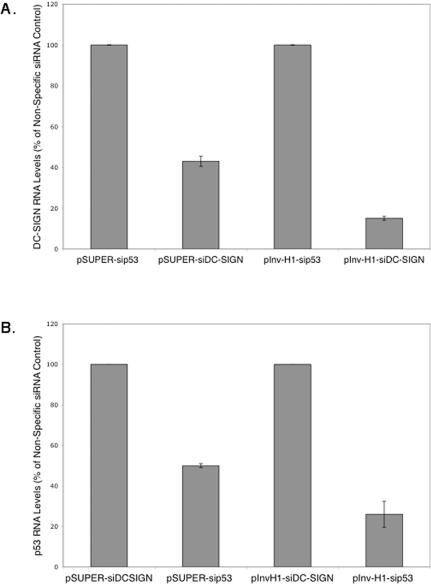
Quantitative polymerase chain reaction (Q-PCR) detecting gene-specific silencing. A. Silencing of DC-SIGN. Total RNA was isolated from 293T cells co-transfected with pcDNA3-DC-SIGN-AU1 and pInv-H1-siDC-SIGN or pcDNA3-DC-SIGN-AU1 and pInv-H1-sip53 and 250 ng analyzed by quantitative RT-PCR. The data presented is from one out of three experiments comparing pInv-H1-siDC-SIGN to that of pSUPER-siDC-SIGN, with DC-SIGN inhibition ranging from 55–85%. Each sample was analyzed in duplicate and standard deviation is shown. B. Silencing of p53. Total RNA was isolated from 293T cells transfected with pInv-H1-sip53 or pInv-H1-siDC-SIGN and 250 ng analyzed by quantitative PCR. The data presented here is from one out of four experiments. Each sample was analyzed in duplicate and standard deviation is shown.

To test the efficiency of pInv-H1, 293T cells were transfected with pInv-H1 targeting a specific gene and the mRNA levels of the target gene were monitored by quantitative polymerase chain reaction (Q-PCR). We first used our construct to silence DC-SIGN. The pInv-H1-siDC-SIGN construct was co-transfected into 293T cells with an expression plasmid encoding DC-SIGN. A significant decrease (85% reduction) in DC-SIGN mRNA levels was observed upon expression of pInv-H1-siDC-SIGN compared to the non-specific pInv-H1-sip53 control (normalized at 100%) (Fig. 2A). The same siRNA sequence was expressed from the pSUPER vector, a construct which gives rise to a short hairpin RNA (shRNA) previously shown to efficiently silence target genes [10]. In this experiment, pSUPER-siDC-SIGN expression resulted in a 60% reduction in DC-SIGN mRNA levels compared to the non-specific pSUPER-sip53 control (normalized at 100%). This indicates that when comparing silencing efficiencies between pInv-H1-siDC-SIGN and that of pSUPER-siDC-SIGN, pInv-H1 silenced expression of DC-SIGN to a greater extent (85% vs. 60% inhibition).

To test the ability of pInv-H1 to silence expression of an endogenous gene, we used our construct to silence p53 expression using a previously validated si-p53 sequence [10]. Upon expression of pInv-H1-sip53, mRNA levels of p53 decreased (80% reduction) compared to the non-specific pInv-H1-siDC-SIGN control (Fig. 2B). pSUPER-sip53 expression resulted in a 50% reduction in p53 mRNA levels compared to the non-specific pSUPER-siDC-SIGN control (normalized at 100%). As was seen with the DC-SIGN siRNA, the pInv-H1-sip53 construct silenced p53 gene expression to a greater extent than pSUPER-sip53. However, we note that the percentage of p53 inhibition with pInv-H1 was more variable from experiment to experiment (25–80% inhibition), and may be related to the differential expression of p53 during different phases of the cell line and/or the variable transfection efficiency between experiments.

### Downregulation of DC-SIGN by retrovirus delivered siRNA

Two major disadvantages exist with the delivery of siRNA by plasmid transfection. The first is that with transfections, siRNA downregulation of the target gene product is transient, limiting the types of experiments that can be done. Secondly, transfection efficiency is often inconsistent leading to significant variations in the level of knockdown. These shortcomings of plasmid transfections have been successfully and efficiently overcome through the use of retroviral or lentiviral vectors that stably express siRNAs [24]–[30]. We decided to examine stable expression of the DC-SIGN siRNA using a Murine Leukemia Virus (MLV)-derived retroviral vector encoding a neomycin phosphotransferase marker gene.

**Figure 3:**
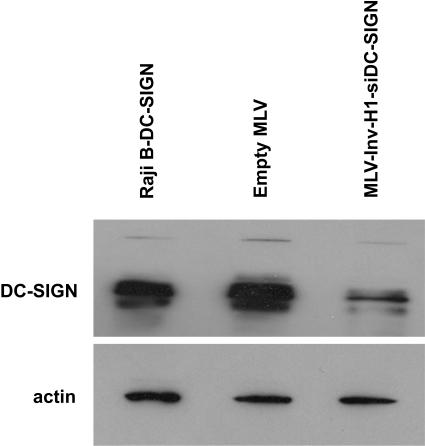
Efficient suppression of DC-SIGN in retrovirally transduced Raji B-DC-SIGN cells. Western blot analysis of DC-SIGN expression in Raji B-DC-SIGN stable cells (lane 1), Raji B-DC-SIGN cells transduced at MOI = 0.1 with an empty retroviral vector (lane 2) or with a retroviral vector encoding the Inv-H1-siDC-SIGN cassette (lane 3). To ensure equal loading of protein, the blot was washed and re-probed for actin.

Raji B cells (a B-cell line) overexpressing DC-SIGN were transduced with either MLV-Inv-H1-siDC-SIGN or an MLV virus lacking the siRNA sequence at a multiplicity of infection (MOI) of 0.1. By using a low MOI, the probability of multiple integrations per cell is reduced, thus transductants are predicted to arise from single integration events. Transduced cells were selected in G418 and expanded. Six weeks post-transduction, we proceeded to evaluate the ability of our Inv-H1-siDC-SIGN cassette, likely present in a single copy per cell, to downregulate DC-SIGN. Figure 3 demonstrates the efficacy of DC-SIGN knockdown at the protein level. Densitometric measurements indicated that DC-SIGN protein expression was reduced by more than 80% in cells transduced with MLV-Inv-H1-siDC-SIGN (lane 3) compared to cells transduced with the parental MLV vector (lane 2). These results indicate that our inverted H1 promoter cassette, even at a single copy level per cell, is capable of maintaining gene silencing 6 weeks after retroviral transduction as these were stable G418 resistant transductants.

## Discussion

In this report, we describe the function of pInv-H1, a vector system that utilizes two inverted minimal H1 promoters to independently drive expression of the sense and antisense strands of a functional duplex siRNA. Although there have been several reports of convergent or tandem promoter systems that function in a similar way, there have been no reports to date of such a system incorporating two minimal H1 promoters. It has been suggested that using two complementary promoter sequences to flank the siRNA-encoding sequence may lead to vector instability [13], [14]. However, we have shortened the minimal promoter unit from ∼300 bp to ∼100 bp and introduced different restriction sites flanking the central siRNA sequence so as to enhance the asymmetry of the promoter cassette and lessen the instability that may come from hairpin formation or homologous recombination.

The initial construction of the entire promoter-siRNA cassette was performed by a ligase chain reaction of multiple oligonucleotides that ranged from 20 nt to 50 nt in length. Once the construct was made, it was easy to exchange siRNA-encoding sequences by simply digesting the vector with *Eco*RI and *Bgl*II and ligating in two annealed oligonucleotides containing the new siRNA-encoding sequence. The shorter oligonucleotides required (45 bp versus 64 bp for the shRNA construct) also makes this vector a more efficient expression vector for multiple siRNAs. Alternatively, we also note that oligonucleotides #7, 8 and 12 (Fig. 1B) can be changed to accommodate any new siRNA sequence; the inverted siRNA expression cassette with the new siRNA sequence can then be made entirely by LCR by re-using the rest of the 11 oligonucleotides that have already been made. This underscores the flexibility of the LCR methodology for generating this minimal inverted H1 promoter, and suggests that additional control elements (such as tet responsive elements or loxP stop sequences) can also be introduced without too much difficulty.

Although the design of pInv-H1 allows for the easy exchange of siRNA cassettes, there is some restriction on the siRNA sequences that can be used in this system. For optimal RNA polymerase III gene expression, purines are required at the 5′ ends of newly initiated RNAs; transcription complexes initiating transcription with pyrimidine residues are less stable than those starting with purines [31]. Therefore, the first base of the 19 nt sequence of the siRNA must be a purine and the last base a pyrimidine for efficient transcription to take place. The siRNA sequences used here to target DC-SIGN and p53 both follow this rule. A direct comparison of the same siRNA sequence produced as a shRNA from the pSUPER vector and a duplex siRNA from our inverted H1 promoter vector suggested that our inverted promoter system is at least as efficient in gene silencing if not more. This may be due to the lack of additional processing required to produce the functional siRNA, as has been suggested by others [14].

We also show that this siRNA expression cassette can easily be shuttled into a retroviral expression system and used to generate recombinant viruses for more efficient cell transduction. Importantly, at an MOI of 0.1, which is likely to result in a majority of single copy transductants, the targeted protein was reduced by greater than 80%. This suggests that the pInv-H1 siRNA expression cassette is highly efficient in mediating gene silencing and may be appropriate for the development of siRNA libraries or siRNA-knockdown transgenic animals. Although inverted or convergent promoter systems for driving siRNA expression have been reported, we show here the application of this system in a retroviral delivery context. By controlling the MOI, we demonstrate that this inverted promoter expression cassette, even at a single copy level, can effectively silence target gene expression.

In summary, we show the generation of our inverted H1 promoter cassette by the use of a ligase chain reaction. The use of the LCR allows for the flexibility of regenerating the entire promoter cassette by substituting a few oligonucleotides. This allows for the potential of “promoter bashing” experiments or including inducible control elements to further expand the capability of our siRNA expression cassette.

## Materials and Methods

### Construction of pInv-H1

To construct pInv-H1-siDC-SIGN, overlapping oligonucleotides were synthesized (MWG Biotechnology; HPLC-purified) and resuspended to 100 µM in water:

InvH1.siDC-S.1: tgcaatCTGCAGATTTGCATgTCGCTATGTGTTCTGGGAAATCACC

InvH1.siDC-S.2: ATAAACGTGAAATgtctttggatttgggaatcTTATAAgt

InvH1.siDC-S.3: tctgtatgaATTcactcaaa

InvH1.siDC-S.4: AGCGAcATGCAAATCTGCAGattgca

InvH1.siDC-S.5: caaagacATTTCACGTTTATGGTGATTTCCCAGAACA CAT

InvH1.siDC-S.6: tttgagtgAATtcatacagaacTTATAAgattcccaaatc

InvH1.siDC-S.7: tctgtatgaATTcactcaaaaac**AATCCAGGCAAGACGCG**

InvH1.siDC-S.8: **AT**gtttttcagtAGATCTatacagaacTTATAAgattccc

InvH1.siDC-S.9: aaatccaaagacATTTCACGTTTATGGTGATTTCCAGAA

InvH1.siDC-S.10: CACATAGCGAcATGCAAATAAGCTTcggctc

InvH1.siDC-S.11: tttgagtgAATtcatacaga

InvH1.siDC-S.12: atAGATCTactgaaaaacATCGCGTCTTGCCTGGATTgtt

InvH1.siDC-S.13: CGTGAAATgtctttggatttgggaatcTTATAAgttctgt

InvH1.siDC-S.14: gagccgAAGCTTATTTGCATgTCGCTATGTGTTCTGGGAAATCACCATAAA

The siDC-SIGN sequence is indicated in InvH1.siDC-S.7 and InvH1.siDC-S.8 in bold. Oligonucleotides 2–5 and 8–13 were 5′-end phosphorylated overnight with T4 polynucleotide kinase (New England Biolabs). Phosphorylated oligonucleotides 2–5 were combined with 6 µl InvH1.siDC-S.1 and 6 µl InvH1.siDC-S.6 (to form LCR-1 product) and phosphorylated oligonucleotides 8–13 were combined with 6 µl InvH1.siDC-S.7 and 6 µl InvH1.siDC-S.14 (to form LCR-2 product) in a ligase chain reaction containing 8 U Pfu ligase (Stratagene) and 5 µl 10× Pfu ligase buffer. The LCR conditions [19] consisted of an initial denaturing incubation of 95°C for 1 min, 40 cycles of 55°C for 1 min 30 s, 70°C for 1 min 30 s, 95°C for 30 s, with an additional incubation at 55°C for 2 min and 70°C for 2 min on the final cycle. The LCR reactions were electrophoresed through a 3% Metaphor agarose gel. Fragments corresponding to LCR-1 and LCR-2 were purified using Quantum Prep Freeze ′N Squeeze DNA Gel Extraction Spin Columns (BioRad Laboratories), ethanol precipitated, and digested with *Eco*RI. The two products were gel purified as described and ligated. The ligation product was digested with *Pst*I and *Hind*III and ligated into *Pst*I/*Hind*III digested pSUPER. To generate pInv-H1-sip53, the pInv-H1-siDC-SIGN was digested with *Eco*RI and *Bgl*II and ligated with the annealed, phosphorylated oligonucleotides corresponding to the sip53 sequence (in bold): forward: AATTCACTCAAAAAC**GACTCCAGTGGTAATCTAC**GTT TTTCAGTA; reverse: GATCTACTGAAAAACGTAGATTACCACTGGAGTCGTTTTTGAGTG.

### Cell culture and transfection

293T cells were cultured in DMEM (Gibco) containing 10% FBS supplemented with 1% penicillin-streptomycin (Gibco). Cells were cultured in 6-well plates and transfected by calcium phosphate. A total of 8 µg DNA was transfected into each well. For DC-SIGN gene silencing experiments, cells were co-transfected with 2 µg pcDNA3-DC-SIGN-AU1 and 2 µg of pSUPER-sip53, pSUPER-siDC-SIGN, pInv-H1-siDC-SIGN, or pInv-H1-sip53. An additional 4 µg of pENTR (Invitrogen), a promoter-less vector used as filler DNA, was also transfected. For analyzing p53 silencing, cells were transfected with 6 µg pSUPER-sip53, pSUPER-siDC-SIGN, pInv-H1-siDC-SIGN, or pInv-H1-sip53 and 2 µg of pENTR.

### RNA analysis

Three days after transfection, total RNA was isolated from cells using Trizol reagent (Invitrogen) according to the manufacturer's recommendations. For each condition, 250 ng of total RNA was analyzed in duplicate by quantitative real-time PCR using QuantiTect RT-PCR reagents (Qiagen). Briefly, RNA was added to QuantiTect probe mix, 3 µM forward and reverse primers, 5 µM probe, 50 mM MgSO_4_, QuantiTect RT mix, and Rnase-free water. A reverse transcription one-step PCR protocol was carried out as follows using DNA Engine Opticon: 30 min 15 sec at 50°C, 10 min at 95°C, followed by 15 sec at 95°C and 1 min at 60°C for 45 cycles. Gene-specific primers and probes for DC-SIGN and p53 were used to quantitate RNA levels. RNA from each sample was also analyzed for actin RNA copies to use as a means for normalization. The primers (MWG Biotechnology) and probes (PE Applied Biosystems) used were as follows: DC-SIGN, 5′-GCTGAGAGGCCTTGGATTCC-3′ and 5′-AGAGCGTGA AGGAGAGGAGTTG-3′, (probe, 5′-FAM-ACCATGGCCAAGACACAC CCTGCTA-TAMRA-3′); p53, 5′-CTGCTCAGATAGCGATGGTCTG-3′ and 5′-TTGTAGTGGATGGTGGTACAGTCA-3′, (probe, 5′-FAM-CCCC TCCTCAGCATCTTATCCGAGTGG-TAMRA-3′); actin, 5′-GCATGGGT CAGAAGGATTCCT-3′ and 5′-TGCCAGATTTTCTCCATGTC-3′, (probe, 5′-FAM-TGAAGTACCCCATCGAGCACGGCAT-TAMRA-3′).

### Construction and Transduction of Retroviral Vector Expressing pInv-H1-siDC-SIGN cassette

The Inv-H1-siDC-SIGN cassette was subcloned into the MLV derived retroviral vector pLIB (Clontech). First, the vector was modified by cloning cDNA derived from pCMMP.GFP/Neo (gift from R. Mulligan) encoding a green fluorescent protein (GFP)-neomycin phosphotransferase gene fusion into the *Sal*I and *Not*I sites of the pLIB MCS. The Inv-H1-siDC-SIGN cassette was subcloned from the pSUPER vector into the *Not*I and *Cla*I sites of the MCS of the modified pLIB vector. The resulting plasmid was co-transfected into 293 cells with MLV gag/pol and VSV-G expression plasmids pMD.gagpol and pMD.G respectively. The resulting viral particles were harvested from the media at 48 and 72 hrs, sterile filtered (0.45 µm) and used to transduce 293 or Raji B cells in the presence of 8 µg/ml polybrene. FACS analysis was conducted 72 hrs post-transduction to calculate viral titer based on the percent GFP positive cells. Viral titers of 1×10^4^ infectious units/ml were obtained.

DC-SIGN overexpressing Raji B cells were transduced using a multiplicity of infection (MOI) of 0.1 in the presence of 8 µg/ml polybrene. Cells were placed in 600 µg/ml G418 48 hrs post-transduction to select for transductants.

### Western Blot Analysis

Six weeks post-transduction, Raji B cells were lysed and total cellular protein was extracted. Protein concentration was determined using the BioRad Protein Assay reagent (Bio-Rad Laboratories). Equal amounts of lysate (35 µg) were resolved on 10% SDS-PAGE and transferred to polyvinylidene fluoride (PVDF) membrane. DC-SIGN was detected using rabbit anti-DC-SIGN antibody and ImmunoPure HRP-conjugated donkey anti-rabbit secondary antibody (Pierce Biotechnology). The blot was developed using ECL Plus (Amersham Biosciences). The blot was washed several times with PBS/0.2% Tween-20 buffer and re-probed with anti-actin HRP (Santa Cruz Biotechnology).
